# When multiple primary lung cancers express the same rare mutation: a case report

**DOI:** 10.3389/fonc.2024.1475193

**Published:** 2024-11-13

**Authors:** Yaqing Han, Yandong Geng, Qian Sui, Yanjie Liu, Shaonan Xie, Maogang Gao, Qingyi Liu, Guangjie Liu, Shize Wang

**Affiliations:** ^1^ Department of Thoracic Surgery, The Fourth Hospital of Hebei Medical University (Hebei Tumor Hospital), Shijiazhuang, Hebei, China; ^2^ Department of Thoracic Surgery, Bao Ding NO.1 Central Hospital, Baoding, Hebei, China; ^3^ Department of Clinical Laboratory, The Fourth Hospital of Hebei Medical University (Hebei Tumor Hospital), Shijiazhuang, Hebei, China

**Keywords:** multiple primary lung cancer, intrapulmonary metastasis, RET mutation, whole-exome sequencing, histological

## Abstract

The debate continues whether the expression of the same rare genetic mutation in multiple primary lung cancers suggests intrapulmonary metastasis or truly multiple primary lung cancers. We report a case of a 54-year-old female patient who presented with multiple nodules in the right lung discovered during a routine examination, persisting for six months. The patient had three central lesions in the right lung’s upper, middle, and lower lobes. She underwent thoracoscopic wedge resection, and the postoperative pathology reported two minimally invasive adenocarcinoma and one adenocarcinoma *in situ*. Interestingly, genetic testing for lung cancer-related driver genes revealed the presence of the rare *RET* mutation in all three nodules. This led us to speculate that these nodules might have the exact origin rather than being multiple primaries. To verify this hypothesis, we conducted further testing on these nodules, including whole-exome sequencing (The NGS data was generated from the Illumina sequencing platform by Novogene Co. Ltd, Beijing, China). The results indicated that although all three nodules expressed the *RET* mutation, there was significant heterogeneity in the gene mutations (differences in the number of cellular mutations, substitution composition levels, and clustering analysis of the three nodules). Thus, the patient was considered to have multiple primary lung cancers. In such cases, whole-exome sequencing can distinguish whether the nodules have the exact origin.

## Background

Lung cancer remains the leading cause of cancer-related deaths worldwide (1). With the increasing application of low-dose CT for lung cancer screening, more cases of multiple primary lung cancers (MPLC) are being detected. The traditional diagnostic criteria for distinguishing MPLC from pulmonary metastasis are based on the Martini and Melamed (MM) criteria (2) and the Comprehensive Histologic Assessment (CHA) criteria (3), which mainly rely on histological comparison.

*RET* gene (proto-oncogene tyrosine-protein kinase receptor *RET*) is a rare genetic mutation in lung cancer, with only 1-2% incidence (4–6). Most MPLC cases show different mutations in different nodules, but some patients’ nodules exhibit the same mutation, necessitating further evaluation to determine whether they are genuinely MPLC.

## Case report

**Figure 1:**
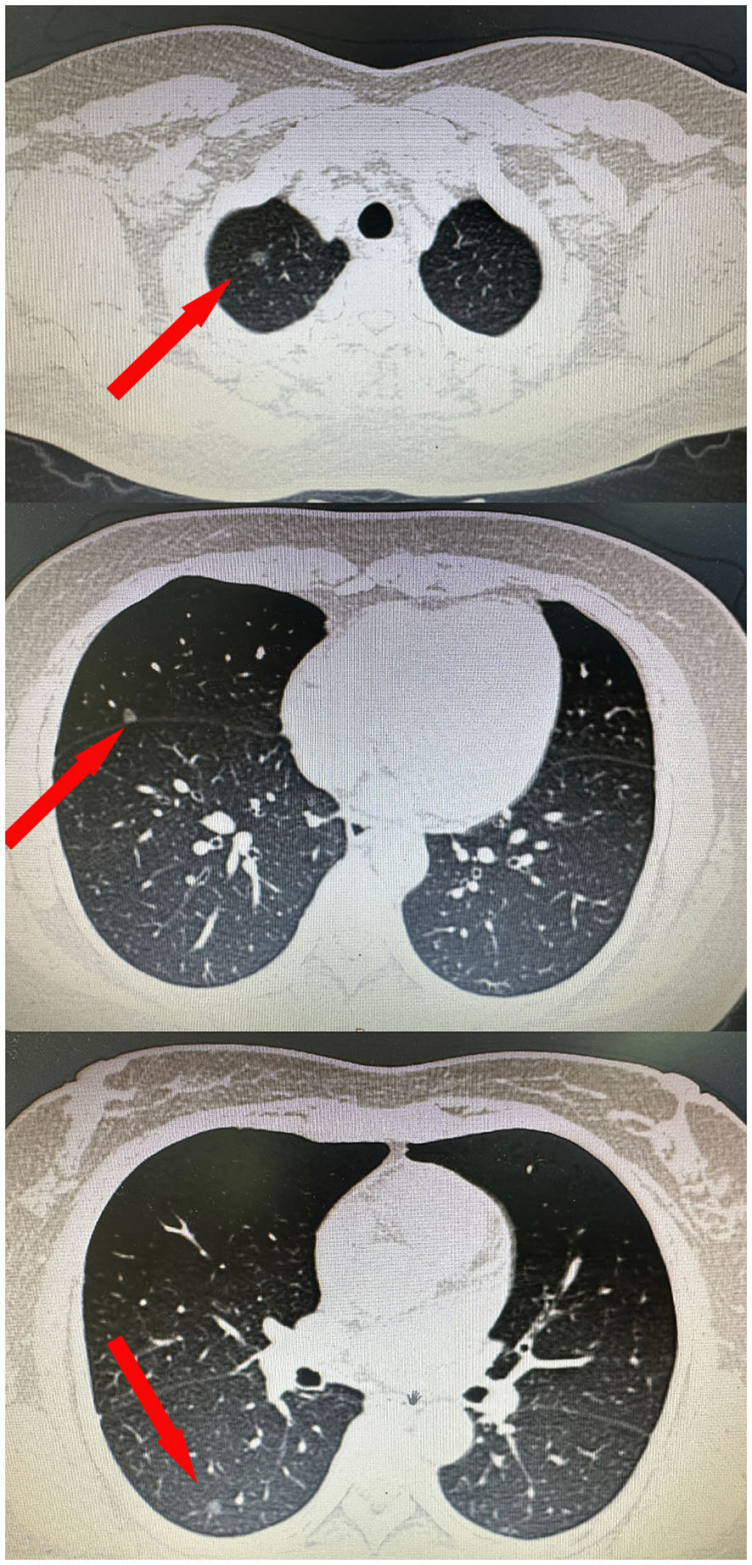
CT data of the patient’s three pulmonary nodules.

The case involves a 54-year-old female patient who sought medical evaluation due to the existence of multiple pulmonary nodules in her right lung over six months. A computed tomography (CT) scan performed at our institution identified three nodules within her right lung’s upper, middle, and lower lobes (**Figure 1**). The postoperative pathological report indicated that the upper lobe nodule was carcinoma *in situ* with a diameter of 0.6cm. The middle and lower lobe nodules were microinvasive adenocarcinomas measuring 0.5cm in diameter. All three nodules were predominantly lepidic, and two microinvasive adenocarcinoma had acinar components. Postoperative genetic examination confirmed *RET* mutation in all three nodules. Considering that *RET* mutation is rare in lung cancer cases, we performed whole exon sequencing (7) on these three nodules to differentiate between multiple primary lung cancers and intrapulmonary metastatic cancer. The results demonstrated varying somatic mutations among the three nodules: 257, 242,105, respectively. Among these mutations, nine were shared, while others were independent. In addition, 6 substitution analysis and 96 substitution analysis showed differences in 3 nodules (t test, p<0.05). The mutation characteristics of the sample and 78 known mutation characteristics were cluster analyzed, and the similarity was found to be low. Based on these findings, we conclude that this patient has multiple primary lung cancers.

## Discussion

Recent years have seen an increase in lung cancer detection due to low-dose CT screening, with over 60% of cases presenting as ground-glass opacities, and up to 10% of patients having multiple ground-glass nodules (8). Most of these cases are considered MPLC (9, 10), although some studies suggest the possibility of metastasis (11). Next-generation sequencing (NGS) is becoming increasingly important in the molecular typing of lung cancer for treatment decisions. Studies have shown that different ground-glass nodules in the same patient often have different gene mutations, and even when nodules exhibit the same gene mutation, their mutation spectra can differ, suggesting the possibility of MPLC. Yanagitani (12) has reported on a 55-year-old woman with advanced lung adenocarcinoma with metastasis in both lungs who showed multiple metastatic ground glass nodules on CT. In recent years, two additional criteria introduced by the American College of Chest Physicians (ACCP) and the International Association for the Study of Lung Cancer (IASLC) emphasize using histological features, immunohistochemistry (IHC), clinical characteristics, and molecular features to differentiate between multiple primary lung cancers and intrapulmonary metastasis (13, 14).

Studies on multiple nodules being primary cancers or lung metastases are limited. Most research is based on targeted genes (9, 10, 15), with few based on genomics (11). Gene targeting for multiple nodules has not found any evidence of intrapulmonary metastasis. Generally, each nodule’s driver gene mutations are highly distinct (9). Next-generation sequencing (NGS) is the most commonly used targeted sequencing technology, allowing for the simultaneous sequencing of millions of DNA fragments and providing sensitive, economical, and high-throughput detection. Therefore, NGS is gaining more and more attention as an auxiliary means in histopathological diagnosis, especially in lung cancer, where molecular typing contributes to treatment selection (16, 17) Research by Park et al. (18) indicated that, by second-generation sequencing of 16 patients with multiple ground-glass nodules, most patients with multiple ground-glass nodules have different gene mutations in each nodule. However, a few patients have the same gene mutation. However, the mutation spectrum is different, suggesting that even if each nodule shows the same gene mutation, the possibility of multiple primary lung cancers still needs to be considered.

Zhou et al. ‘s (19) study showed that whole exon sequencing could distinguish multiple primary tumors from intrapulmonary metastases, including CNV(Copy Number Variation) analysis, somatic mutation, 6 substitution analysis, 96 substitution analysis and other methods. This study suggests that pulmonary metastasis exists in multiple nodules, but the number of multiple nodules is not related to the probability of pulmonary metastasis. Other genomic profiling studies (20–22) have also reported sizeable genetic heterogeneity among multiple lesions of MPLC. Applying genomic analysis may be crucial for accurately managing patients with multiple nodules.

**Figure 2:**
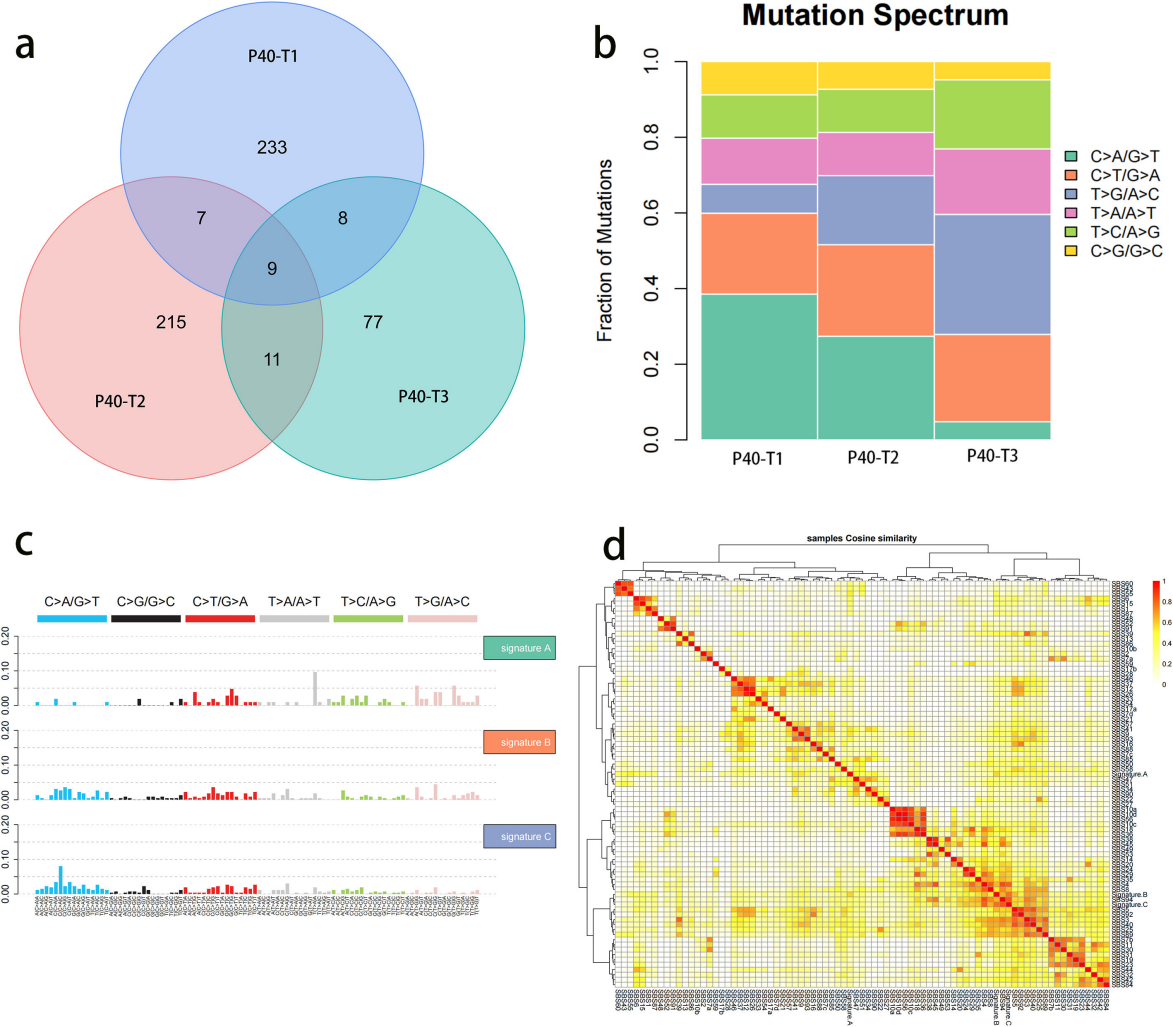
Analysis of total exon results of three nodules. **(A)** Number of somatic mutations in three nodules. Colored circles represent different nodules, and the intersection is the common mutation of the three nodules. **(B)** The abscissa in the mutation spectrum histogram is the sample name, the ordinate is the proportion of each mutation type in the sample, and different colors represent different SNV types. **(C)** Proportion of each mutation feature in different samples. The abscissa represents the sample, and the ordinate represents the proportion of each mutation feature. **(D)** Cluster analysis of the mutation features of samples and 78 known mutation features. The darker the color is, the closer the cosine value is to 1, signifying that the higher the feature similarity is, the more likely it is the same feature.

The *RET* gene is a proto-oncogene located on the long arm of chromosome 10. It encodes for a tyrosine kinase receptor and is involved in the proliferation, apoptosis, and invasion of tumor cells, affecting the development and progression of tumors (4). The formation of tumors is primarily associated with *RET* gene fusions, which account for only 1-2% of lung cancers. This report presents a unique case where a patient’s CT scan revealed three ground-glass nodules, considered to be multiple primary lung cancers. Postoperative pathology reported that the nodule in the right upper lobe was a 0.6 cm *in situ* carcinoma, the nodule in the right middle lobe was a 0.5 cm minimally invasive adenocarcinoma, and the nodule in the right lower lobe was also a 0.5 cm minimally invasive adenocarcinoma. However, postoperatively, genetic testing revealed that all three nodules expressed the *RET* mutation, an occurrence with an extremely low probability. We conducted whole-exome sequencing to assess further whether the patient’s nodules were intrapulmonary metastases. The results of whole exon sequencing showed (**Figure 2**) that the number of somatic mutations in the three nodules differed, 257,242,105, respectively, and only 9 were common among the three nodules. In contrast, other mutations were independent of each other. However, the mutations in the single nucleic acid showed that the proportion of mutations in each nodule was different, and the mutation spectrum of different nodules in patients was significantly inconsistent at the level of substitution composition (t-test,p<0.05). The mutation features of the three samples were clustered with 78 known mutation features in the COSMIC website, and the three mutation features were not similar to any of the known 78 features (cosine similarity <0.9), the cosine similarity between the three mutation features being less than 0.9.The similarity is not high. Therefore, we conclude that this patient has multiple primary lung cancers.

This case report indicates that for patients with multiple lung cancers, each nodule exhibits independent genetic characteristics, including in cases of rare mutations. The case presented here involved three nodules, all *RET* mutations, with an incidence of less than one in a hundred thousand. Through whole-exome sequencing analysis, it was shown that despite the presence of the same mutation in all nodules, genetic heterogeneity still exists among them. Genetic testing cannot be used as a basis to distinguish between multiple primary lung cancers and intrapulmonary metastasis, but further differentiation can be achieved through whole-exome sequencing.

## Data Availability

The datasets presented in this study can be found in online repositories. The names of the repository/repositories and accession number(s) can be found below: https://www.ncbi.nlm.nih.gov/, PRJNA1000732, https://www.ncbi.nlm.nih.gov/, PRJNA1000739.
